# The Voluntary Hospital System and the Proposed Ministry of Health

**Published:** 1918-10-26

**Authors:** 


### BRITISH HOSPITALS ASSOCIATION CONFERENCE.

### The Voluntary Hospital System and the Proposed Ministry of Health.

A well-attended conference of members of the Association was held on Friday, October 18, at St. Thomas's Hospital, under the presidency, in the absence of Lord Sandhurst, of Mr. H. Wade Deacon (Treasurer of the Liverpool Royal Infirmary).

Those present included Sir George H. Makins, G.C.M.G., representing the Ministry of Health Committee of the Colleges of Physicians and Surgeons, the Hon. Sir Arthur Stanley, G.B.E., Sir Henry Burdett, K.C.B., Mr. H. Wade Deacon, Col. Montefiore, Rev. G. B. Cronshaw, M.A., Mr. Edwin J. Layton, Mr. T. Hall Hall, Mr. C. E. Leo Lyle, Col. E. M. Bruce Vaughan, Mr. N. Bishop Harman, F.R.C.S., Major W. McAdam Eccles, F.R.C.S., Mr. Thos. J. Whiffen, Major T. Basil Rhodes, R.A.M.C., Canon Graves, Col. J. E. Broadbent, C.B., Mr. T. G. Greek Wills, Col. Sir John Rutherford, Bart., M.P., Rev. B. W. Bradford, and representatives of hospitals and institutions in various centres, viz. :—

**London.**—Sir James Harrison, Major S. Weil, Messrs. G. A. Arnaudin, G. Cunningham, Alexander Pym, H. J. Campbell, Edward J. Blackett, R. Cramp, G. Panter, F. W. Drewett, Harold Wigg, E. M. Williamson, Sidney M. Quennell, Louis H. M. Dick, R. T. Lawlor, Frank Sordy, L. Gray, Walter Kewley, J. A. Jennings, W. G. Martin, A. H. H. Franklyn, P. Michelli, C.M.G., F. N. Kay Menzies, Jas. M. Churchfield, E. W. Morris, Ludovic G. Foster, Chas. T. Walrond, W. J. Davidson, Arthur Watts, Godfrey H. Hamilton, J. Richardson, Herbert H. Jennings, A. L. Harrison, P. J. M. Rogers, Edwin Fox, G. J. Furness, F. Stanley Cheer, Lady Barrett, Misses A. E. Cummins, Constance Tatton, Margaret Tatton, Marion Ritchie.

**Provincial.**—Messrs. T. N. Webb (Truro), W. Banks and Gerard H. Smith (Derby), A. Griffiths (Ipswich), B. Brooks and E. C. Smith (Birmingham), Thomas Maddock (Wellingborough), Fredk. Gore (Margate), Sydney Morris and Frank Inch (Norwich), Thomas H. Gregg (Bristol), Roden Orde (Newcastle-on-Tyne), F. Ransom (Hitchin), C. P. Lovelock (Carshalton), Frank G. Hazell (Manchester), J. H. A. Marchand (Barnet), J. H. Shaw (Southport), W. H. Head (Cambridge), C. S. Risbee (Northampton), J. W. Barnes (Sheffield), C. R. W. Offen (Leamington), W. H. Harper (Wolverhampton), Arthur Preston (Guildford), Capt. J. Arthur Cowley (Northwich), Frank Oliver (Ashton-under-Lyne), Miss R. Hooper (Coventry), the Hon. Secretary of the British Hospitals Association, Mr. J. Courtney Buchanan.

The CHAIRMAN reminded members that a special committee was appointed some time ago to watch the Ministry of Health Bill when introduced, and to take such action as they might think necessary. Though the Bill had not yet been introduced, the committee asked its secretary, Mr. Buchanan, to present a statement of the position of the voluntary hospitals, and to make certain suggestions. The committee and Council met, and decided to circulate the pamphlet which Mr. Buchanan had prepared among the members of the Association and ask their views. The circular had brought no specific suggestions, but there had been general approval of the pamphlet and of the general principles enunciated in it. Whatever views might be held on the subject, members would feel greatly indebted to Mr. Buchanan for the trouble and pains he had taken in compiling the pamphlet. (Applause.) The general underlying principle was a very simple one—namely, the maintenance of the voluntary principle. There would, of course, be grounds for differences of opinion and for discussion, but he thought it would be wise to avoid debating details to-day. So far as was known, the proposed Bill did not directly affect voluntary hospitals at all, though its indirect effect upon them might be very considerable. Some of the propositions stated in Section VII. would probably meet with general approval. The Council had invited Sir George Makins to propose a resolution, and the Rev. G. B. Cronshaw to second it.

Sir GEORGE MAKINS, G.C.M.G., proposed: "That the British Hospitals Association welcomes the proposal to form a Ministry of Health. The Association is strongly of opinion that the interests of the patients and of the community as a whole, the progressive education and training of doctors and nurses, and the prosecution and advance of scientific research will be best served and provided for in the scheme by the retention of the voluntary hospital system."

He said the resolution dealt with two questions. The first was the mere question of the formation of a Ministry of Health, and it could be said at once that the proposal to form such a Ministry was meeting with the support of the nation and the medical profession alike. The interests of this meeting were mainly situated in the future of the voluntary hospital system. That system was one of the great links with the past of which the nation generally had most reason to be proud. It had endured for many centuries, and through great national upheavals. One had only to look at the institution in which the meeting was now assembled after the great upheaval in the time of Henry VIII. and the loss of the hospital's charter at that date. The nation felt the value of the institutions of this class to an extent which obliged the next monarch, Edward VI., to restore the charter and a portion of its property. The voluntary hospital system as it existed now had formed the basis on which the whole hospital system of this country was founded, and it was one of which we might justly be proud. Moreover, the voluntary hospitals had been the home, in this country, of medical training, and they had produced the medical practitioner of the day and exerted a paramount influence on his training, taking the practical part in making him a highly-trained man. The Universities had taken an interest in fostering medical learning. It had been said that a central administration of medical schools would tend to a general uniformity and to increased efficiency. Admittedly something was to be said on that side. But, on the other hand, it must be recognised that, while it might tend to a general efficiency and uniformity, it would also tend to reduce every institution to the same level—possibly a dead level which was cramped and hampered by official administration. A change of that kind would remove that proper spirit of emulation between the hospitals and the schools which had done so much in the past to help progress and to advance medical teaching and medical treatment. One great feature of medical education in this country had been the individuality of the teaching in the separate schools. There were differences in the kind of teaching given at them, and a strong spirit of emulation, each striving to take a foremost place, with a resultant advance all along the line. If all students were turned out under exactly the same system, he did not think it would be good for the medical man of the future. During a somewhat long life as a teacher and examiner he had had some practical experience of this, and he had always felt that he had gained much knowledge and valuable experience from having come into contact with students from various schools. In his opinion, in the country's present emergency a considerable proportion of the success which had attended the Medical Services had depended on the fact that the men who had done the work had been gleaned from all quarters, and men with views which were often somewhat divergent had had to

**British Hospitals Association Conference**—(*cont.*)
work together in concert and to some extent in emulation. That, he considered, had had a very wholesome influence on the medical work of the war as a whole.

There was another side of the work of the voluntary hospitals which one had to take into consideration, a side which, he thought, would be lost at once were all these hospitals to be masssed under one central administration. He referred to the social side. There were many important problems now awaiting solution, problems in which the public were taking greater and greater interest. One referred to the work being done by the so-called hospital almoner, *i.e.* the continuous care of and interest in the patient independently of his actual stay in the hospital, so that the benefit gained by the stay in hospital was not lost. There was also the question of baby welfare and maternity work. He supposed the voluntary hospitals had done more, at any rate in London, to improve the maternity work than had any other influence which had been brought to bear. Also the same agencies had taken a very prominent part in getting the treatment of tuberculous patients put upon a proper basis. That was largely because these hospitals were individual institutions, free to undertake any enterprise which seemed to them to be fitting and suitable. It seemed clear that many things would be undertaken under such conditions which probably would not be taken up by a great central administration. The present system gave an opportunity of individual effort and trial which, he thought, might result in the solution of problems which otherwise might hardly have been taken up, or, at all events, which might have been delayed in their solution for a long period.

Another side was the influence which a hospital exerted upon the patient, a matter which was first brought very much to the front by Miss Nightingale as the result of her experience here and of hospitals in war-time. He felt that a hospital which was under the control of a voluntary committee manifested a different spirit from that of a hospital which was run by a Government Department, and there would be a great loss in that way if a transference were to take place to a central Department. And what would be the result of such a change? At present voluntary hospitals owed much of their succcess to the very strong sympathy existing between the general public and the aims and the work of the hospitals; it was looked upon as a charitable work. He thought we ought to regard the expression "charitable work" in the religious sense—namely, not as a question of receiving money, but as one of doing good to others. In the event of a transference of the management of these hospitals to a Government Department he thought the whole of that would be lost. He did not think anyone could fully appreciate what degree of influence was exerted upon the country generally by the existence of this feeling of sympathy between the public and the work being done by the hospitals. It was a channel by which sentiment was developed into action, and, after all, most of the best, the most far-reaching, things done in the world were accomplished under the influence of sentiment. He, as a servant of the voluntary hospital system of some fifty years' standing, felt strongly that it would be a disaster if the voluntary hospital system were to collapse, and for that reason it gave him very great pleasure to propose this resolution, inadequately though he had been able to deal with the subject. (Loud applause.)

The Rev. G. B. CRONSHAW, in seconding the resolution, said the chief surgeon of Radcliffe Hospital, Oxford, who was also doing much military work, remarked to him that the work he did in that hospital gave him the greatest satisfaction of any he did; he felt he had there the means of doing absolutely the best for the patient, as no request was ever denied. Not one at that meeting would wish that spirit to disappear. Moreover, there was not the tortuous channel through which requisitions had to pass in a Government-controlled institution. Any obstacle to hospitals keeping up with the latest advances would be a retrograde step. The voluntary hospital principle in this country was the envy of the whole world. America was now trying to get back to it after a trial of the State administration of hospitals; New Zealand and Australia had got a sort of half-way house between the two, and had found the voluntary system the best for the country. Those concerned with voluntary hospitals in this country had got to set their houses in order; they would need to do something more for the country than in time past. Realising that legacies were diminishing, that a former £50 legacy had to be made good by fifty subscriptions of £1, it would be seen that help had to be secured, and that without disturbing the voluntary principle. Hospitals had now more beds to provide than they thought possible in the days gone by. He was often asked why it was only the very poor had the best services of specialists and the up-to-date remedies, and why the middle classes could not share in these good things. These latter could not afford the specialists' fees, but they could pay something. He thought this insistent cry could be met without diminishing the principle underlying the work. Those responsible for the hospitals would welcome the creation of a Ministry of Health to ask managers what the voluntary hospitals could do to make the nation better. The Prime Minister had referred to the difficulty of building up an A1 nation on C3 men. It was the business of hospitals to help provide the A1 men. It could be done with a little more forward policy and more openness of vision, and to deal with one central Ministry instead of separate authorities according to the nature of the work would be a great advantage. He welcomed a system by which every doctor in the neighbourhood of a hospital would be free to follow his patient into it, and not only give the help required, but also procure what was most needed for him. Money could be accepted from public funds while still preserving the voluntary hospital principle. In the matter of the National Insurance Act the voluntary hospitals had stood between the nation and the complete collapse of that Act. He looked forward to a future when they could do more for the patient (therefore for the country), and more for the doctor and nurse, for he looked for the time when nurses engaged on child welfare and other special work would be allowed, under certain conditions, to re-enter their hospital to bring their knowledge and practice up to date.

The CHAIRMAN called attention to the proposal that steps be taken to educate the general public as to the importance of their own personal interest in their local hospital, and the benefits accruing from their being run on voluntary lines.

Sir HENRY BURDETT, K.C.B., said he was anxious to speak first, because rather important information came to him that morning from the United States which, he thought, might have a considerable bearing on this Association's future. He was quite in agreement with the two former speakers; what they had said must commend itself to all in the room. Arising out of the war, the United States Government had come to the conclusion that the Hospitals Association of America was a very important body, and that the work being done by the hospitals, many of which were voluntary, was worthy of being strengthened. And so it had come about that there was to be granted what we should call the American equivalent of a Royal Charter. H.M. the King did not yield to anybody in his interest in hospitals. Before the war he set aside a definite time every year for visiting them and to render them personal service. This Association must take a very high and important position if it was going to be of the use in the rebuilding of the nation it ought to be. He felt that this would be a fitting time to start it on its larger journey by the grant of a Royal Charter. He recommended this view to the meeting and to the Council. He felt that, arising out of the new Department, there would have to be the payment of the hospitals' medical staff: he did not think there could be any doubt about that. Mr. Cronshaw had referred to the skill and science of hospitals being avail-

**British Hospitals Association Conference**—(*cont.*)

able in future for all, and the demand would make necessary the admission into hospitals of people of every class for varied payments. When the Duchess of Connaught was very ill in Canada she went into a hospital, and owed her recovery largely, under Providence, to the fact. Catering by the hospitals for all grades of the population could not come without a system of payment of what was at present the honorary staff. After fifty years of experience and of very grave consideration of the economic side of the question, he considered that if all the medical men in hospitals were to be paid to-morrow it would prove, in practice, an economic measure which might surprise his hearers. That was a decision to which, he thought, the members of the medical profession were gradually coming. They had been very dissatisfied, and with good cause. Wherever it had been necessary to make provision for a new department of a hospital for which some payment had been given it had almost invariably been inadequate, because it had left out the fact that the work had to be done by the members of the medical profession, who were supposed to give extra time beyond all that which they had been giving before as members of the staff for practically nothing. The matter seemed ripe for full consideration and acceptance. He invited his hearers to examine into the administration of hospitals as it was thirty years ago and as it is to-day. Grasp the enormous growth and cost of everything, including the *personnel*, the enormous development and extension, how many members of the staff they had, and the expenditure they worked hospitals on. Let them then take the number of departments for the cure and care of disease, and the number of persons engaged in that work. Let them look at a splendid hospital like St. Thomas's, where they had probably the best system of almoners' work in the whole country. It would be found that, through the extreme skill, ability, and devotion of the lady who had been the head, and largely the maker, of that department, this hospital was in the proud position of taking more part in the welfare and happiness and daily life of the people than the general population had any idea of. Few things were done in the poorer households of an unusual character in the area from which the patients of St. Thomas's Hospital came which did not entail a call on the almoner, and which did not at once find a solution of the difficulty with cheerful help. That was a great thing from the hospital point of view, but it was a much greater one for the citizens, and he knew nothing more touching and cheering, or which made them thank God and take courage for the future of the hospitals, than the work done through the almoner's department when it was adequately organised and thoroughly kept up to its work by conscientious workers. In truth the voluntary hospitals had been the saviours of our working population and the protectors of the poor. Those who knew the Continental methods were seized with this knowledge, and its apprehension had spread to members of the general population. The voluntary hospitals must endure as a great bulwark of the nation's freedom and safety.

One further point he wished to urge was that a teaching hospital should have a medical superintendent, a matter which must be fought out with the profession. In the United States it had been gone into very thoroughly, and the course pursued was the result of conviction. The conclusion was that there should be a medical superintendent who should be responsible for the administration throughout, but should have nothing to do with finance. The latter was the layman's work. Inside the hospital it was essential to have a man who was educated and trained, one of the best type of medical men of the day, in charge. He pointed to the extraordinary development of the work done by the members of the American Hospitals Association, which had gone up enormously since this system was extended some twelve years ago. The medical superintendent in America was paid more than he probably would have earned in the ordinary pursuit of his profession. In view of there being other speeches to follow, he had not entered into details, but he wished to draw attention to three important and material points which he felt sure were now ripe for solution and must come with the changes which this meeting had been called to discuss. (Applause.)

Mr. COWLEY (Northwich) said the strong feeling in his town was that the voluntary system must remain. He suggested that resolutions passed at this meeting should be sent to every voluntary hospital in the country, and that boards of management of hospitals should be asked to pass similar resolutions and have them distributed to every hospital subscriber. He would also enlist the active co-operation of the various Hospital Saturday committees.

Major LOVELOCK (Carshalton) thought the provision of private wards would be the answer to many of the difficulties, especially financial ones. His own hospital had six such wards, and they were always full, the patients paying a very fair sum weekly. Probably one-third of the hospital income came from these wards. Recently a committee had been sitting in his town to consider the Ministry of Health proposals, on which practically every section of the community was represented. It decided that the voluntary system must remain, but that the new Ministry must look after those parts of the country in which there was a lack of hospital accommodation or where the finances of existing hospitals needed strengthening.

Mr. MARCHANT (Victoria Hospital, Barnet) endorsed the view that the co-operation of the great hospital funds should be sought.

Mr. WILLS (South Devon Hospital, Plymouth) felt sure that if working men were consulted more in hospital matters it would be much better for hospital committees. That had been followed at Plymouth, and with very good results.

Mr. JENNINGS (Chelsea Hospital for Women) thought the speaker had wandered somewhat from the main purpose of the meeting, which was to get public opinion to bear on the Ministry of Health as to whether the voluntary system should be maintained or not. How best could the needed propaganda work be carried on?

Mr. BOULTBEE BROOKS (Queen's Hospital, Birmingham) said that, although the voluntary system was one of which they were truly proud, times had now changed, and demands on hospitals had greatly increased. He did not see, with Sir George Makins, why, under central control, hospitals should sink to a dead level. Why should they not all rise to one high standard? ("No.") Hospitals should continue to be managed in the voluntary manner, but they should be prepared to receive grants-in-aid, either direct from the State or through the town or city councils. Representation of working men already obtained in many hospitals in the provinces.

Mr. SMITH (Warneford Hospital, Leamington) urged that successful hospital propaganda work benefited not only the hospital, but, reflexly, the town too. The work of a hospital should be made thoroughly known in the town. People should be shown that the hospital was being run in their interests, and be taught to regard it as their hospital.

Mr. N. BISHOP HARMAN (West London Hospital) pointed out that the establishment of a Ministry of Health would mean bringing all the Poor-Law institutions into its purview, and these comprised fully two-thirds of all the hospital beds available. These would no longer be under the stigma of the Poor Law. And alongside these would be the voluntary hospitals. That seemed to him the most serious new situation which would have to be faced; the relationship which one set of hospitals would bear to the other set. There would, he thought, be real competition. He supported the plea for voluntary as against State institutions, and quoting the parallel case of national education, he did not see why the receiving of help from the State should alter the voluntary principle of management. The King's Fund was concerned with London only, and he suggested that, at this juncture, the voluntary hospitals should be thrown upon the mercy of the Charity Commissioners.

**British Hospitals Association Conference—(*cont.*)**

Major W. McADAM ECCLES (St. Bartholomew's) said there were two main principles to be borne in mind. The first was that our national health should be improved. Secondly, in this improvement and in the maintenance of our medical and nursing education there was a first place for our voluntary hospitals. The latter had all the experience of the past to guide them, which State institutions had not got, not even the Poor-Law infirmaries. He believed that when the Government came to look at this matter of a Ministry of Health they would realise that the voluntary hospitals were a magnificent asset in this country. But every effort should be made to bring them up to standard, so that the Ministry could say, "These are good, let them remain." He urged the fostering of visits to the hospitals by all kinds of people, so that they could see the work which was being carried on there.

Sir ARTHUR STANLEY, G.B.E., M.P., thought he could safely say, on behalf of the Council of the Association, that the discussion had served the purpose which it was meant to. No one had any doubt that a Ministry of Health would come, and another matter on which there was not likely to be disagreement was that the voluntary hospitals had got to remain. The meeting was called because the Council wanted to get suggestions and a clear lead on certain definite points from the various hospitals, not the big London hospitals so much as the hospitals generally throughout the country. He did not think that for a long time to come the voluntary hospitals had anything to fear from a Ministry of Health. Dr. Addison told him, in conversation some time ago, that those who were in charge of the Bill for the Ministry of Health had no intention of trying to enforce any particular principle or doctrine of their own; all they wanted to do, in the initial stage, was to collect under one roof and into one Ministry all the powers affecting public health which were now exercised by many different Departments. From what he knew of public Departments and of the complexity of questions concerning public health, he would say that the new Ministry had its work cut out for a good many years to come. (Laughter.) He thought the people of the country would always insist on having the voluntary system alongside the State system for the control of health. A very good parallel was that of the R.A.M.C. and Red Cross Services. Nothing could be better as a State Service than the R.A.M.C.; that had been proved in this war. Our Army in this war had been better looked after than had any Army in the history of the world. But alongside the R.A.M.C. was a body built up by the public, and insisted on by them, the Red Cross, not only to supply the little extra comforts and luxuries which meant so much to sick and wounded men, but one of the great functions of the Red Cross was to keep up the stimulus all the time. The public made certain, by having an independent body of its own, that the standard of efficiency of the treatment given was that which ought to be given. There was no better instance of that than what happened in Mesopotamia. When the Government learned how bad the conditions there were they set themselves to remedy them, and promptly asked the agencies which voiced the opinion of the public, the Red Cross and the Order of St. John. He believed that the value of the work of those bodies did not lie so much in the fact that they could get launches and hospital ships and equipment out there much quicker, but in the fact that through those bodies the light of public opinion was brought to bear on the actual conditions in that part of the world.

What now remained to be done was to see that the case for the voluntary hospitals was put in the best possible way, both inside Parliament and in the country generally. He was glad to say that this Association could now claim to represent practically the whole of the voluntary hospitals of the United Kingdom, so that when a meeting such as this was summoned it could be relied upon to represent the opinion of the whole of those institutions. That was a great step forward in view of what Mr. Bishop Harman said about the beds of the Poor-Law hospitals, which were well represented in the House by the President of the Local Government Board and others. The procuring of a Royal Charter for the Association had been suggested. A Royal Charter was a veritable two-edged weapon: one he had particularly in mind had been a big nuisance. But if the Charter were properly drawn, and supported by people who knew what they wanted, it was a very useful instrument, and he would like to take the suggestion from this meeting to the Council. He also thought they should get a number of members of Parliament interested in their case, and fully equipped with all the essential information. He suggested that the more freely as he did not propose to be in the next Parliament himself. But he had often seen a good case spoiled by the lack of a good advocate. He would like to see the Association appoint a small Parliamentary body to look after its interests when the Ministry of Health Bill came to be discussed. (Applause.) He was certain the Association had got a good and popular case, and that those concerned with voluntary hospitals all agreed on the main lines. Mr. D. SMITH, Derby, and Mr. DREWETT also spoke.

The resolution was carried unanimously.

The following resolution, duly moved and seconded, was likewise carried: "That the Council be requested, when the terms of the Ministry of Health Bill are known, further to consider the question with reference to its provisions; to take such steps as, at their discretion, they may determine; and report."

The meeting concluded with votes of thanks to the Governors of St. Thomas's Hospital for generously placing the room at the disposal of the Association, and to Mr. Deacon for presiding.

### Voluntary Hospitals and the Proposed Ministry of Health Bill.

We are asked to state that practically the whole of the second edition of this pamphlet, issued by the British Hospitals Association in August last, has been sold. If any voluntary hospitals require further copies for their Committees they must order at once, as the type must shortly be distributed.

